# Impact of antiplatelet therapy and cardiopulmonary bypass on platelet function in patients undergoing coronary artery bypass *grafting* using multiple electrode aggregometry

**DOI:** 10.1186/1749-8090-8-S1-P63

**Published:** 2013-09-11

**Authors:** B Gersak, M Petricevic, K Praprotnik, B Biocina

**Affiliations:** 1 School of Medicine, University of Ljubljana, Ljubljana, Slovenia; 2 Department of cardiac surgery, KBC Zagreb, Zagreb, Croatia

## Background

Antiplatelet therapy (APT) is known to substantially reduce mortality and rate of ischaemic complications after coronary artery bypass grafting (CABG). Rate of perioperative APT resistance varies widely and could be influenced by cardiopulmonary bypass (CPB). The purpose of the study was perioperative assessment of platelet function with respect to administered APT and CPB, and determination of patients with APT resistance who could benefit from more aggresive treatment strategy.

## Methods

In prospective study we enrolled 192 patients undergoing elective CABG. Patients were divided into 4 groups with respect to their preoperative APT management. All patients received Aspirin (ASA) 300 mg/ day postoperatively starting on the day of procedure. Platelet function was assessed prior to surgery and at fourth postoperative day (POD 4) using multiple electrode aggregometry (MEA). Adenosine diphosphate (ADP test) and arachidonic acid (ASPI test) induced platelet aggregation tests were used.

## Results

Group of patients exposed to ASA preoperatively had lower values of ASPI test (P<0.001) comparing to patients not receiving ASA. However, we registered 28.6% ASA resistant patients. Both ASPI (P<0.001) and ADP (P<0.001) test values increased significantly at POD 4, suggesting postoperative platelet hyperactivity. Postoperatively, we registered 33.3% ASA resistant patients despite higher ASA dosing regimen.

## Conclusion

MEA can recognize patients with ASA resistance during the both the pre- and post- CABG period. Postoperatively, ASA 300mg did not sufficiently inhibit platelet aggregation in 33.3% patients. In this subgroup, dual antiplatelet therapy with ASA and clopidogrel could be useful for maintaining graft patency, and preventing adverse ischemic events.

